# Q&A: What are strigolactones and why are they important to plants and soil microbes?

**DOI:** 10.1186/1741-7007-12-19

**Published:** 2014-03-31

**Authors:** Steven M Smith

**Affiliations:** 1 ARC Centre of Excellence in Plant Energy Biology, University of Western Australia, Crawley 6009, Western Australia

## What are strigolactones?

Strigolactones are signaling compounds made by plants. They have two main functions: first, as endogenous hormones to control plant development, and second as components of root exudates to promote symbiotic interactions between plants and soil microbes. Some plants that are parasitic on other plants have established a third function, which is to stimulate germination of their seeds when in close proximity to the roots of a suitable host plant. It is this third function that led to the original discovery and naming of strigolactones.

## Where does the name come from?

**Figure 1:**
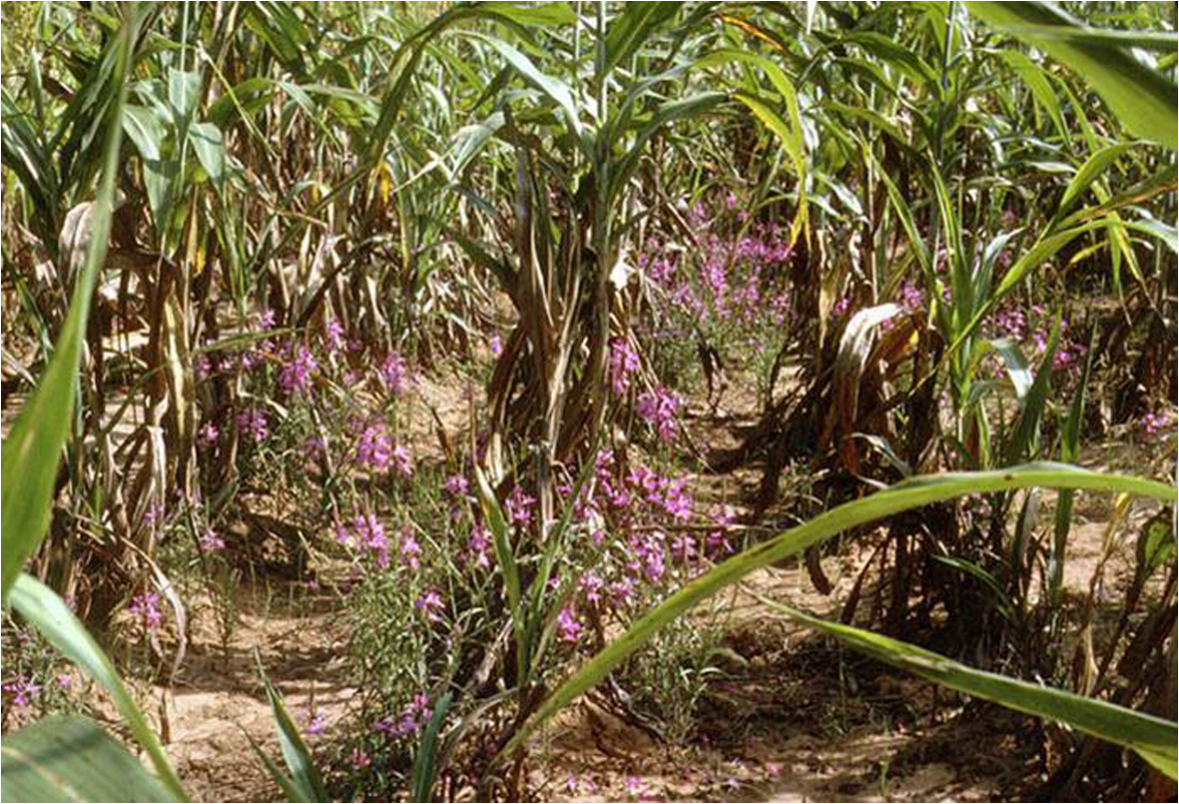
**Plants of the witchweed *Striga hermonthica *parasitizing maize plants in Africa.** Photo reproduced by permission of L J Musselman , taken from [2].

Strigolactones were discovered in root exudates due to their ability to stimulate germination of seeds of the parasitic plant *Striga*, the ‘witchweed’ [1]. One example of this family of plants is *Striga hermonthica*, the purple or giant witchweed (Figure 1). So where did *Striga* get its name from? Witchweeds were so-named by subsistence farmers in Africa because they appeared without warning apparently from nowhere, and attacked their crops. The scientific (Latin) name for these witchweeds derives from Striga, a mythical witch apparently with origins in ancient Rome but known in several parts of southern and central Europe. The witch Striga was thought to be filled with hatred towards others, especially children, feeding on their life essence, or consuming them without remorse. *Striga* species are members of the broomrape family (Orobanchaceae), most members of which are parasitic on other plants.

**Figure 2:**
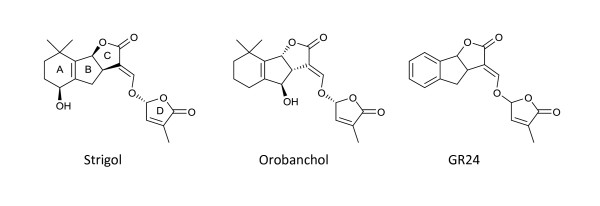
**Strigolactone structures.** Strigol and orobanchol are naturally occurring, and their stereochemical structures are shown. GR24 is a synthetic analogue and shown without stereochemical configuration because it is synthesized as a racemic mixture. The lettering convention for ring structures is shown on the strigol molecule, where C and D are both lactones.

The lactone part of the strigolactone name refers to the chemical structure. In chemistry, a lactone is a cyclic ester - the condensation product of an alcohol group and a carboxylic acid group in the same molecule. In fact, strigolactones have two lactone rings (Figure 2). Members of the strigolactone family differ in the chemical modifications to the core structure and in their stereochemical (three-dimensional) conformations. Thus, strigol and orobanchol are two common examples in which the A and B rings, respectively, are oxidized, and in which the stereochemistry of the B ring relative to the C ring is different (Figure 2).

## Why do plants secrete strigolactones if they stimulate attack by parasitic weeds?

**Figure 3:**
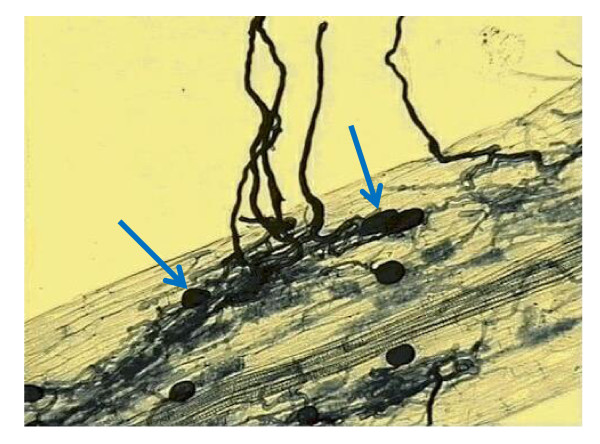
**Arbuscular mycorrhizae in clover root, showing arbuscules (blue arrows) within the root cortex, and hyphae radiating from the root surface.** Image taken from [4]. Courtesy of Jim Deacon, The University of Edinburgh.

It was discovered in 2005 [3] that strigolactones (at least the synthetic strigolactone GR24) stimulate hyphal branching in a fungal symbiont that forms arbuscular mycorrhizae (AM) on their host plants. Arbuscules are complex structures that form inside the cortical cells of the plant root (Figure 3) and it is thought that the strigolactone effect on hyphal development helps the fungus to colonize the host root and to form arbuscules. This intimate association of fungus and root benefits the plant because the fungal hyphae spread widely in the soil to acquire mineral nutrients, especially phosphate and nitrate, which the plant then takes up. The fungus benefits by obtaining carbon and nitrogen metabolites (energy and amino acids) from the plant. Up to 80% of all plant species form mycorrhizae. Plant mutants that are defective in strigolactone production or exudation are also impaired in their ability to form AM. It appears, therefore, that strigolactones are exuded by plant roots specifically to promote the association with these symbiotic fungi.

Plants in the broomrape family have exploited the fact that other plants exude strigolactones to use them as signals to trigger germination of their seeds. Since strigolactones will only be present in close proximity to plant roots, the seeds that germinate will immediately attach to the roots to start the colonization process. Thus, these parasitic weeds are opportunistic, and in evolutionary terms are ‘newcomers on the block’ relative to the symbiotic soil fungi.

## Do strigolactones have any effect on the plant that makes them?

**Figure 4:**
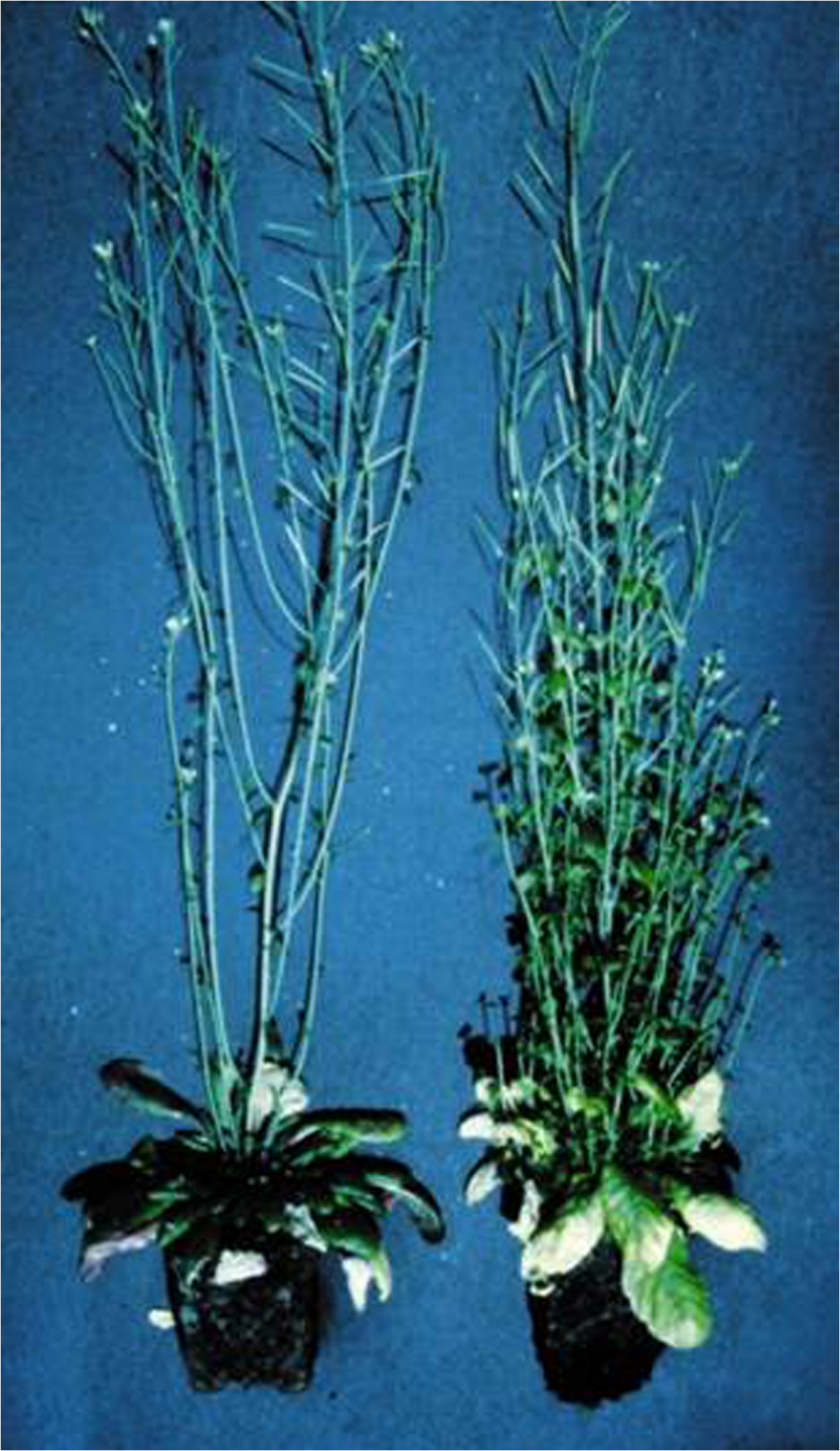
**The strigolactone-deficient mutant of *Arabidopsis *shows exuberant branching.** The wild-type plant (left) has few secondary shoots compared to the mutant (right). Figure reproduced with permission from [8].

Yes, strigolactones influence many aspects of plant development. This first became apparent in 2008 [5,6] when it was discovered that a previously unidentified chemical signal transported from roots to shoots to repress the outgrowth of secondary shoots is actually a strigolactone. The evidence came from observations that plant mutants unable to make strigolactones produced many secondary shoots (Figure 4), and these could be prevented from growing by applying the synthetic strigolactone GR24. Subsequently, it has been discovered that strigolactones induce secondary thickening of the stem, and can promote the formation of lateral roots and root hairs [7].

The result of such effects of strigolactones is that the root system grows in preference to the shoot system. Why? The answer is that it helps the plant to scavenge for mineral nutrients in the soil, while at the same time conserving the resources of the plant. The advantage of this becomes clear when we realize that strigolactone production is increased in response to nutrient limitation in the soil. So when soil nutrients are scarce the plant invests resources into finding more, instead of using limited resources to grow the shoot. Remember too that strigolactone production in the roots will encourage the formation of arbuscular mycorrhizae - another strategy to acquire minerals from the soil. Conversely, when mineral nutrients are plentiful, strigolactone production will decline, less will be transported to the shoot and new secondary shoots will grow to increase the capacity of the plant to capture energy from the sun and carbon dioxide from the atmosphere.

## Strigolactones have several functions - which came first?

Strigolactones can be traced back to some simple single-celled algae and primitive land plants such as mosses and liverworts [9]. Their original function was presumably in signaling between cells and in the control of growth and differentiation in early plants. For example, strigolactones are found in mosses, liverworts and in the alga *Chara coralline,* where they promote rhizoid growth. The filamentous moss *Physcomitrella patens* produces strigolactones that can regulate protonema branching and growth of filaments of a neighboring colony [10]. Thus, we see how growth and competition of neighbors can be coordinated by strigolactones - a principle that operates within higher plants to coordinate root and shoot growth. With colonization of the land several hundred million years ago, came fungal symbioses. Some liverworts enter into symbiotic relationships with mycorrhizal fungi, and although we do not yet know if this interaction depends on strigolactones, it is a hypothesis worthy of testing. With the evolution of vascular plants came complex patterns of shoot branching and the opportunity for long distance transport of strigolactones. It is in the flowering plants that the important functions of strigolactones are best known and best understood. The exploitation by witchweeds of strigolactones exuded by host plants is the latest invention in the evolutionary history of strigolactones.

## How are strigolactones made by plants?

**Figure 5:**
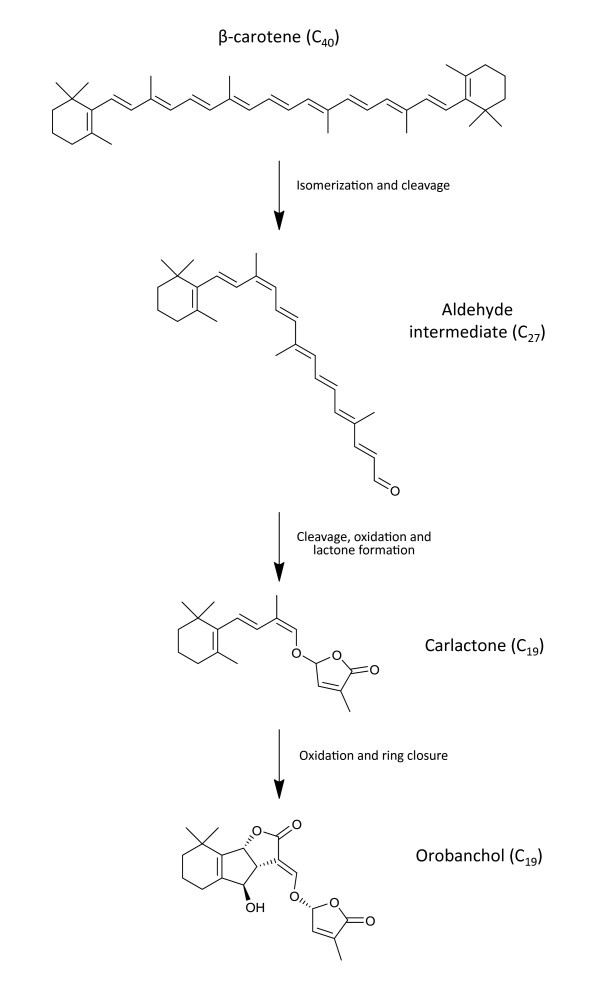
**Simplified biosynthetic pathway from β-carotene to orobanchol.** The steps from β-carotene to carlactone are believed to require only three enzymes: one isomerase and two carotenoid cleavage dioxygenases, all of which are found in the plastid. The subsequent conversion to strigolactones such as orobanchol is less well understood but apparently occurs outside the plastid and requires a cytochrome P_450_ enzyme.

Strigolactones are made from carotenoids, which in turn are made from building blocks called terpenes or isoprenes. Carotenoids and hence strigolactones can therefore be described as terpenoids or isoprenoids. While carotenoids provide the yellow, orange and red pigments that you see in banana fruits, carrot roots, and tomato fruits, they have other important functions, including a key role in photosynthesis where they absorb light energy and protect the photosynthetic apparatus against oxidative damage. Carotenoids are also precursors of abscisic acid, a hormone that controls the response of plants to environmental stress. The biosynthetic pathway to strigolactones has recently been shown to involve three chloroplast enzymes that convert beta-carotene to a lactone, given the name carlactone [11] (Figure 5). This is then oxidized in the cytosol of the cell to produce strigolactones. More recently an ATP-dependent transporter protein has been discovered that transports strigolactones out of the cell, either for long distance translocation within the plant or for exudation from roots [12].

## How are strigolactones detected by plant cells?

Plant hormones are invariably detected by a receptor protein, which triggers interaction of that protein with other proteins to elicit a signal transduction cascade leading to changes in cell activity. The strigolactone receptor was identified through studies of mutants that are insensitive to strigolactone treatment, including the rice *dwarf 14* (*d14*) and petunia *deceased apical dominance 2* (*dad2*) mutants. Isolation of the *D14* and *DAD2* genes showed that they encode members of the α/β-barrel family of proteins with strong similarity to esterases [13,14]. The proteins are able to hydrolyse the D-ring of GR24, but very slowly. Crystal structure analysis of these proteins has revealed the products of D-ring hydrolysis in the active site of the protein, and small conformational changes compared to the protein in the absence of strigolactone [15,16]. Mutation of a key serine residue in the active site of the esterase renders the protein inactive. It is believed that conformational changes in the D14-type protein can mediate its interaction with other proteins in the cell to elicit strigolactone responses.

## How is the strigolactone signal transduced into a response?

Strigolactone hydrolysis by D14-type proteins promotes their interaction with an F-box protein named D3 in rice, or MAX2 in *Petunia* and *Arabidopsis*. This in turn targets other proteins for tagging with ubiquitin, which marks the protein for destruction. Arguably the most critical one is D53, discovered in rice [17,18]. This protein is necessary for the outgrowth of lateral shoots or tillers, most likely through the regulation of gene transcription, but in the presence of strigolactone it is tagged with ubiquitin by the D14-D3 complex, and destroyed [19]. Thus, strigolactones maintain a brake on the growth of new lateral shoots. We can speculate that such a mechanism acting on proteins similar to D53 might regulate the growth of lateral roots, but this remains to be determined. A recent separate study has provided evidence that the *Arabidopsis* MAX2 protein targets the transcriptional regulator BES1, a positive regulator of signaling by the brassinosteroid plant growth hormone, for degradation. This degradation of BES1 is promoted by D14 and strigolactones [20]. However, not all responses to strigolactones are mediated through changes in gene expression. Strigolactone has been found to trigger depletion of the auxin transporter PIN1 from the plasma membrane of xylem parenchyma cells in the stem within 10 minutes of treatment, before any changes in gene expression [21]. Thus, there are probably several mechanisms by which strigolactones regulate cell function and hence plant development.

## How are strigolactone signals integrated with other signals to control growth?

**Figure 6:**
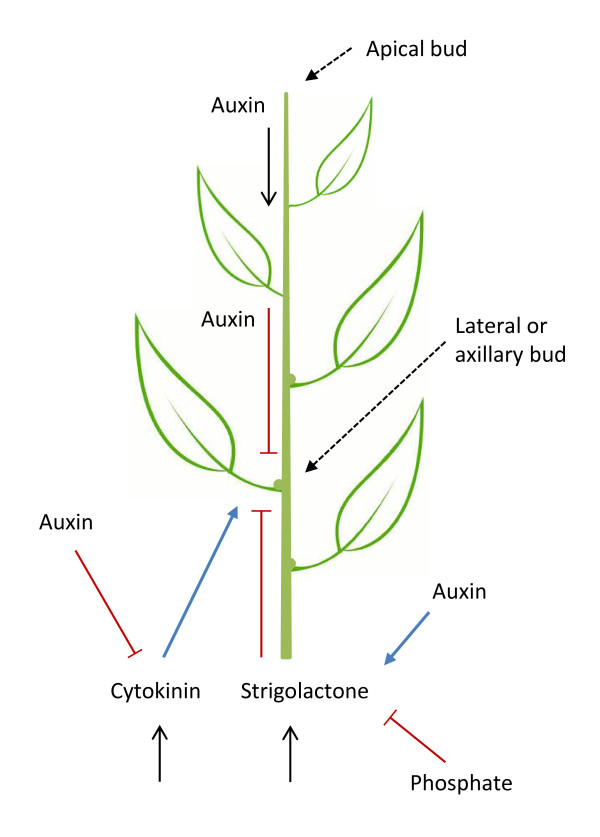
**Interactions between hormones in the control of lateral shoot growth**. Auxin is transported from the apical meristem while cytokinin and strigolatone are transported from the roots (shown by black arrows). Red bars show repression while blue arrows show activation. Lateral buds have the potential to grow into side shoots when the hormone balance permits, and can be achieved experimentally by removing the apical meristem.

Lateral bud growth is inhibited by auxins transported down from the shoot apex and by strigolactones transported upwards from the root. However, cytokinins, also transported from root to shoot, can promote bud outgrowth [22]. These different signals are modulated in response to different environmental factors, such as light and nutrients, and are integrated through crosstalk between biosynthesis and signaling pathways (Figure 6). For example, auxins can stimulate expression of strigolactone biosynthesis genes and repress those of cytokinins, thus serving to reinforce the inhibitory effect of auxins on bud growth [7]. The recent observations of Wang *et al*. [20] also point to potential interactions between strigolactone and brassinosteroid signaling. Although brassinosteroids do not directly influence shoot branching, the strigolactone-dependent destruction of BES1 could potentially dampen brassinosteroid signaling. In a similar vein, yet another study suggests that D14 exhibits strigolactone-dependent binding to the gibberellic acid (GA) signaling protein SLR1 [23]. This could provide a means for strigolactones to modulate GA signaling, which promotes seed germination and stem elongation in many plants.

## Going back to symbiotic fungi, how do they detect strigolactones?

We know that cells of arbuscular mycorrhizal fungi can detect strigolactones because they respond to them, but we have no idea how they detect them. There is no reason to suppose that the mechanism is the same as that in plants. Indeed, the D14-type proteins that recognize strigolactones in plants are not found in fungi, yet similar proteins can be found in *Bacillus*. Treatment of arbuscular mycorrhizal fungi with GR24 induces changes in mitochondrial function, but we do not know if this is a direct or indirect effect of the GR24 treatment [24]. This is an area of importance for future research, since the management of plant-fungal symbioses is so important to ecosystem wellbeing.

## Why are strigolactones so important in agriculture?

The Green Revolution gave us high-yielding dwarf cereals that are highly productive in intensive agriculture systems employing fertilizers and pesticides. Such dwarf varieties have reduced production of or sensitivity to gibberellins so that the plant invests less energy in stem growth and more in seed production. World supplies of phosphate are finite and nitrogen fertilizers are made in large amounts from fossil fuels, so in the future we will need new crop varieties that use nutrients more efficiently. We may also need to rely more on symbiotic relationships between plants and soil microbes to promote plant growth. Genetic variation in strigolactone responses could provide an opportunity to breed plants with superior nutrient use efficiency and ability to form symbiotic associations. In particular, strigolactone control of root and shoot architecture could be exploited to breed better plants [19]. At the same time we can look for opportunities to minimize parasitism by witchweeds, particularly in Africa where they cause severe losses to subsistence farmers. This might be achieved by plant breeding, or by designing superior strigolactone analogs that can be used to stimulate suicidal germination of witchweed seeds in the soil, before the crop is planted. Strigolactone research, therefore, has a very important future to help address some key challenges in crop breeding and management.
